# An analytical perspective of Global health initiatives in Tanzania and Zambia

**DOI:** 10.1186/s12913-016-1449-8

**Published:** 2016-07-18

**Authors:** Aziza Mwisongo, Alice Ntamwishimiro Soumare, Juliet Nabyonga-Orem

**Affiliations:** Health Systems and Services Cluster, World Health Organization Regional Office for Africa, B.P. 06 Brazzaville, Congo

**Keywords:** SWOT analysis, GHIs, Africa, Harmonisation, Alignment, HSS

## Abstract

**Background:**

A number of Global health initiatives (GHIs) have been created to support low and middle income countries. Their support has been of different forms. The African Region has benefitted immensely from GHIs and continues to register an increase in health partnerships and initiatives. However, information on the functioning and operationalisation of GHIs in the countries is limited.

**Methods:**

This study involved two country case studies, one in Tanzania and the other one in Zambia. Data were collected using a semi-structured questionnaire. The aims were to understand and profile the GHIs supporting health development and to assess their governance and alignment with country priorities, harmonisation and alignment of their interventions and efforts, and contribution towards health systems strengthening. The respondents included senior officers from health stakeholder agencies at the national and sub-national levels. The qualitative data were analysed using thematic content analysis in MAXQDA software.

**Results:**

Health systems in both Tanzania and Zambia are decentralised. They have benefitted from GHI support in fighting the common health problems of HIV/AIDS, tuberculosis, malaria and vaccine-preventable diseases. In both countries, no GHI adequately made use of the existing Sector-wide Approach (SWAp) mechanisms but they largely operate through their unique structures and committees. GHI efforts to improve general health governance have not been matched with similar efforts from the countries. Their support to health system strengthening has not been comprehensive but has involved the selection of a few areas some of which were disease-focused. On the positive side, however, in both Tanzania and Zambia improved alignment with the countries’ priorities is noted in that most of the proposals submitted to the GHIs refer to the priorities, objectives and strategies in the national health development plans and, GHIs depend on the national health information systems.

**Conclusion:**

GHIs are important funders of health in low and middle income countries. However, there is a need for the countries to take a proactive role in improving the governance, coordination and planning of the GHIs that they benefit from. This will also maximise the return on investment for the GHIs.

## Background

Global health Initiatives (GHI) are a new form of partnership providing support to countries for development [1]. There are various definitions of GHIs most of which characterise GHIs by a set of common features, including their focus on an issue of international concern, operation in several countries, substantial funding, performance-based inputs, and direct investment in countries [2–4]. A number of GHIs were created to support low and middle income countries [1]. This new funding mechanism arose from the need to advocate for, mobilise and hasten funding for some key health problems facing the globe [5]. Over the past 20 years, approximately 100 or so GHIs have been created with the aim of assisting countries to achieve their health outcomes in line with the global goals [1, 5]. GHI support has been of different forms, ranging from monetary support for certain underfunded priority areas to technical support to improve health support systems for their better performance [4]. GHIs are also renowned for prompting the involvement of civil societies in planning and advocating for services [6], for being pro-poor and for supporting areas that affect the poorest of the poor. Furthermore, GHIs have been instrumental in promoting innovations meant to ease implementation of interventions [1].

GHIs have been a subject of contestation for several reasons [4, 5, 7–9]. At the pinnacle of these debates is the use of the tem term itself. For example, Nervi [2] claims that several initiatives that self-identify as global are in fact bilateral in nature, involving only one recipient country. Regarding their management, among the contentions surrounding GHI governance is that they are characterised as vertical, extremely demanding, and burdensome for the limited human resources in the low and middle income countries [4, 10]. Several studies point out that GHI management approach tends to duplicate efforts, is poorly coordinated and harmonised, and lacks country ownership [1, 3, 6, 11]. The nature of the support rendered by GHIs also has been a subject of debate [6, 10] particularly their level of funding, peculiar schedules and strict conditions and restrictions, which are perceived to water down the positive synergism of countries’ funding efforts [4]. Evidence further highlights the concerns over the implementation of GHIs in countries and how they impact the addressing of the countries’ priorities, long-term investments and sustainability of interventions [4].

The African Region has benefitted immensely from GHIs and continues to register an increase in their number. Their assistance has helped Africa to make tremendous progress towards the achievement of some key global health goals such as the Millennium Development Goals (MDGs) [4].

Countries with low levels of health expenditure have been able to advance interventions for priority health problems mostly through GHIs funding [8]. Some of the MDGs have been achieved through GHI support for key interventions such as immunisation, prevention of mother to child transmission of HIV, HIV related interventions, case detection of tuberculosis and provision of insecticide treated bed-nets [8, 12]. GHIs have been able to do this through their ability to mobilise huge levels of financial resources, linking inputs to performance and channelling resources directly to non-governmental civil society groups for greater accountability [7].

The mushrooming of GHIs has resulted in the complexity of the aid architecture [1], partly because of the limitations in the centralised information on all GHIs and their operations. Information on how they function and operate at the country level and their contribution to the harmonisation and alignment of partner programmes with country priorities and health systems strengthening is scarce and outdated. Most of the literature on GHIs focuses on their initiation phase; documentation on current developments is limited.

To fill the gaps in information, we conducted country case studies in Tanzania and Zambia. The main objectives were to understand the profile of the GHIs supporting health development in the two countries and assessing their governance structures, alignment with country priorities, harmonisation with other GHIs and donors, and contribution towards health systems strengthening.

## Methods

In this article we adopt the definitions of the health system as provided by the World Health Organisation (WHO) defining a health system as consisting of “all organizations, people and actions whose primary intent is to promote, restore or maintain health”[12] organised around six building blocks namely service delivery; health workforce; information; medical products, vaccines and technologies; financing; and leadership and governance (stewardship) [13]. Health system strengthening is on the other hand defined as “improving these six health system building blocks and managing their interactions in ways that achieve more equitable and sustained improvements across health services and health outcomes”[14].

The operation definition for GHIs applied in this study was based on the following characteristics as identified by Shiffman and Smith [15];

- An organised effort linking people and organisations
- Involves several countries
- Addresses a major health issue of international concern
- Focuses on specific diseases or on selected interventions, commodities, or services
- Has the ability to generate substantial funding
- Its inputs are linked to performance
- Involves direct investment in countries
- Involves partnerships with nongovernmental organisations and civil society

With this definition, the GHIs considered in this study include (1) those that are involved in research and development in product discovery, and development of new diagnostics, drugs and vaccines, e.g. the GAVI Alliance; (2) those that render technical or service support with the intention of improving service access, such as those that provide discounted or donated drugs; and (3) those that provide financing for specific disease programmes and health systems strengthening.

### Study design and focus

Country case studies in Tanzania and Zambia were used to assess the operations and dynamics of GHIs in Africa. The assessment concentrated on their modalities of financial support, areas and scope of support, and influence on health systems, including service delivery in key health priority programmes. For in-depth understanding of health systems strengthening (HSS), the HSS framework based on the six World Health Organization (WHO) building blocks (Table 1) was applied in the analysis. This framework is operationalised by Warren et al. [10] to enable detailed assessment of health system investments. It allows comprehensive quantification of system-level interventions, as indicated in Table 1.

**Table 1:** WHO building blocks HSS core and specific areas

Building Block	Specific functions
Governance	Capacity building Harmonisation Sector integration Decentralisation National health strategy development Coordination
Financing	Maximise social protection Improve resource effectiveness Patient and/or provide incentives Financial management
Information	Health information strengthening systems Strategies to increase evidence-based planning Increase accessibility of information
Human resources	Support for pre-service training Support for in-service health workforce
Medicines and technology	Support for rational use of essential medicines Improve management of essential medicines Affordable, quality essential drugs programme Health service supplies (non-consumables)
Service delivery	Infrastructure Measures to increase coverage-supply Measure to increase coverage-demand

### Country selection, and study participants

The case studies were undertaken in Tanzanian and Zambia. These countries were purposely selected taking into with consideration their economic grouping based on World Bank estimates for gross domestic product per capita, the presence of GHIs and other partners supporting the health sector, language, and level of investment from GHIs. In each country the study population was drawn from government agencies, GHI staff at national and sub-national levels, civil society organisations (CSOs), international partners at the national and sub-national levels and frontline implementers of GHI programmes at the national and sub-national levels. An attempt was made to achieve as broad a range of perspectives as possible.

### Data collection and analysis

#### Semi-structured interviews

Twenty semi-structured interviews were carried out at the national level with members of the Harmonization for Health in Africa (HHA) committees, GHI officials, and representatives of the Ministry of Health and the treasury, and of key CSOs. An initial list of all the key stakeholders was developed with the help of HHA coordinators at WHO offices in each country. HHA is a collaborative initiative of key multilateral organisations and donors who provide support to governments in health systems strengthening. The snowballing technique was then used to add other respondents as they were identified by stakeholders. The respondents were senior officials who were involved in either partnership structures or development and implementation of health programmes in the broader health sector or implementation of GHIs. The draft questionnaire was pretested with health systems staff at the WHO Regional Office for Africa and adjusted prior to undertaking the survey.

The interviews sought the views of respondents on the profile of GHIs, their governance and alignment with country priorities, harmonisation of their interventions, and efforts and contribution towards health systems strengthening. Before the interviews, the respondents were briefed in detail on the purpose of the study. The interviews were administered by a consultant using a semi-structured questionnaire. On average the interviews lasted 45 min.

#### Document review

Several documents were reviewed to assess the GHI situation in each country, particularly literature on GHI policies and guidelines, operating procedures and strategic plans. The goal was to ascertain which GHIs were operating in the country, the areas they supported, their governance structures and their contribution to health systems strengthening.

#### Group interviews

In each country one district was selected taking into consideration a number of factors including support of its interventions by at least two GHIs, convenience of travel or time constraints and existence of either good or worst case studies on GHI investment.

In each district one focus group of 12 respondents consisting of senior or mid-level managers was conducted. Selection of the group members was based on their roles and involvement with GHIs in the chosen districts. Group discussions sought to collect respondents’ views on the profiles of the GHIs, their governance and alignment with country priorities, harmonisation of their interventions, and their efforts and contribution towards health systems strengthening.

#### Data analysis

Qualitative information was transferred into MAXQDA software. Data were analysed deductively, emerging issues were identified and coded in line with both pre-determined themes, in line with the study objectives. Identified themes were then compared across the participants and the two countries.

## Results

### Tanzania and Zambia’s health system contexts

At independence in 1961 Tanzania developed a national health system that committed the country to provide the mostly non-urban population with access to health services. To meet the health needs of the rapidly growing, largely rural population, the government structured the health system so that ill people were referred from a local first point of contact to increasingly specialised, more central facilities. That multi-tiered, decentralised health system continues to operate to this day.

The ongoing process of decentralisation by devolution in Tanzania is stretching the managerial staff capacity to coordinate activities across the different ministries and fulfil their roles within the Ministry of Health and Social Welfare and the Prime Minister’s Office, which is in charge of the regional administration and local government structures. The health system includes the national level as the overall policy-maker and the regional, district, ward and community levels (where there are health posts) as the implementation levels.

Health systems weakness in Tanzania relate to shortage of human resource with an estimated 0.52 health care workers per 1,000 population, below the WHO recommended 2. 28 per 1000 population [16]. Government per capita expenditure on health is estimated at only US$13 while donor funding plays a significant role [17].

In 2012 the Zambian government modified the roles and functions of its two main ministries dealing with health. The Ministry of Health was assigned the role of policy- and decision-making for secondary and tertiary hospitals, curative care and training schools, while the Ministry of Community Development, Mother and Child was mandated with the control of primary health care levels and the responsibility of ensuring integration of interventions at that level. To facilitate efficient and effective coordination of activities, sector coordination structures were established at three administrative levels:

- Provincial Health Offices (PHOs) are responsible for coordinating health service delivery in their respective provinces,
- District Health Offices (DHOs) are responsible for coordinating health service delivery at district level, and
- At community level, neighbourhood health committees were established to facilitate linkages between the communities and the health system.

Although the Zambia health system has registered some improvements in the previous 3 years, it still suffers gaps in the different components of the health system [18]. For example, government percapita expenditure on health is only US$46 below estimated requirement to finance a minimum package of health services. Available human resource is still below the WHO recommended minimum threshold [18].

#### GHI governance

Tanzania is renowned for its large number of foreign aid partners in comparison with other African countries. Like many low and middle income countries, it has seen an influx of GHIs since 2000 supporting its strategic efforts to fight of HIV/AIDS, malaria and tuberculosis. The prominent GHIs are the US President’s Malaria Initiative (PMI); the US President’s Emergency Plan for AIDS Relief (PEPFAR); the Global Fund to Fight AIDS, Tuberculosis and Malaria (GFTAM); the GAVI Alliance; the Roll Back Malaria partnership; UNITAID; Stop TB Partnership; and the Global Leprosy Programme.

Some of the prominent GHIs in Zambia are GFTAM; the GAVI Alliance; PMI; PEPFAR; the Global Alliance for Improved Nutrition; Children’s Investment Fund Foundation; the Measles Initiative and the Global Polio Eradication Initiative.

In Tanzania, GHIs commonly have their own governance structures operating through committees they specifically create to serve their interests. For example, GFATM operates through country coordinating mechanisms that comprise a range of stakeholders, including public and private CSOs. The GAVI Alliance uses an interagency coordinating committee to oversee and make decisions on proposals and support. This committee has representation from both public and private entities. Other GHIs such as the Roll Back Malaria Partnership and Stop TB Partnership operate through United Nations agencies. All United States based GHIs are housed under the Center for Disease Control and Prevention (CDC).

Many of the GHIs in Zambia, including GFTAM and the GAVI Alliance, do not have offices in the country but operate through committees that are mandated to plan for and determine the priorities for grant proposals. Other GHIs operate through multilateral agents like WHO. All United States based GHIs have staff responsible for programme planning and implementation at the national level. In both countries, none of the GHIs uses existing SWAp mechanisms for planning for and financing of the health sector, but they operate through their own structures, which was a concern, as noted by one of the respondents,

*SWAp is a very good structure that has been in place for many years now. However, these strong GHIs do not make use of it … Like we are here in this meeting preparing our annual plan but they are not participating.* (MoH officer, Zambia)

#### National health strategic plans and national priorities

In Tanzania, the third health sector strategic plan that was published in 2008 by the Ministry of Health and Social Welfare is the key policy document for the health sector for the period July 2009–June 2015. It serves as the guide for planning at council and hospital levels for the achievement of the national goals of the national programme for economic growth and poverty reduction and the Millennium Development Goals. It includes 11 strategies related to health service delivery, covering district health services; referral hospital services; central level support; human resource for health (HRH); health care financing; public–private partnerships; maternal, new-born and child health; prevention and control of communicable and non-communicable diseases; emergency preparedness and response; social welfare and social protection; and monitoring, evaluation and research.

Zambia’s national health strategic plan 2011–2015 refers to a set of strategies for the development of the health sector over 5 years. It serves as an overarching policy framework for all health service activities within the broader framework of the national policy set out in the sixth national development plan 2011–2015, and the national decentralisation policy (2003). The national health strategic plan is operationalised through the medium-term expenditure framework and annual activity-based budgets.

#### Perceptions of GHIs contribution to NHSP by Tanzanian and Zambian stakeholders

In Tanzania, most of the stakeholders perceived the GHIs to be aligned with the third health sector strategic plan and to have played a major role in the control of epidemics communicable diseases such as HIV/AIDS. However, some stakeholders remarked that service delivery approaches by the GHIs contributed to inequity among the population owing to prioritisation of a few diseases and areas. Others believed that the GHI contribution to the national strategic plans was only partial, since often their services were not as comprehensive as required in the strategic plans.

Development partners in Zambia have been supporting the implementation of the national health strategic plan by providing resources to the MoH through the expanded health and human resources baskets and through sector budget support. They also support the poverty reduction budget through un-earmarked funds for the Ministry of Finance and National Planning. All the stakeholders believed that the GHIs were aligned with Zambia’s national priorities, since they bought into the country’s development plan, they used the country’s national health strategy as a guide and they collaborated with district health teams in implementation of activities. One of the MoH officers noted that,

*Development partners through SWAp are dedicated to support Zambian development plans. We plan together and make sure that we do not duplicate activities*.

In both countries the stakeholders indicated that most of the proposals submitted to the GHIs referred to the priorities, objectives, strategic directions and programmatic approaches of the national health development plans. This can be of great value in enhancing coherence between proposals and the countries’ medium- and long-term plans if the health development plans are new or updated. Often, however, the existing national health development plan is old with most of its strategic elements outdated. In such cases, reference to it could result in discrepancies between the proposals submitted to GHIs and the country’s real situation. This indicates the need for government authorities to ensure that their national health development plan is current.

#### GHI contribution to health system strengthening

### Governance

In Tanzania, some GHIs such as PEPFAR have been involved in strengthening the management capacity of health leaders at the sub-national level in the effort to address the weaknesses they have noticed in governance. This has involved capacity building for better planning, accountability and performance through a training programme called WAJIBIKA. However, according to the respondents, these efforts are limited and unsustainable, as governance is a government responsibility and needs to be based on a clear road map. Such a road map does not exist in Tanzania, and evidence shows that the Tanzanian government is hesitant to develop it.

Following the misappropriation of funds at the MoH in Zambia that was revealed by the Anti-Corruption Commission and the forensic audit by the Office of the Auditor General, the management of GFATM was moved from the government to the United Nations Development Programme in 2009. This was an interim arrangement as efforts were being made to improve MoH governance and financial and procurement management. A detailed governance plan was developed supported by the Global Fund, donors and other GHIs. The features of the plan were outlined as:

#### Development of the governance plan

The Government of Zambia developed a governance action plan to help strengthen internal control systems at the Ministry of Health and restore confidence in it in the short and medium terms, while substantial changes would be defined following the implementation of a full systems audit. The action plan was developed by the government together with partners. The plan included the following interventions:

- Build capacity within procurement, accounts and internal audit units;
- Re-establish and strengthen the role of the audit committee;
- Undertake a systems audit of the accounting, auditing and procurement functions;
- Strengthen transparency and accountability in financial management;
- Strengthen checks and balances systems in the flow of funds;
- Streamline the accounts structure of government;
- Strengthen oversight of the use of resources in the sector (resource allocation steering committee);
- Take legal action on suspected fraud.

Progress made in the implementation of the plan includes:

- The integrated financial management system and Navision system have been installed and are operational;
- Financial management, accounts, internal audit and procurement systems have been strengthened;
- Positions and reporting arrangements for the accounts, internal audit and procurement units have been realigned to ensure greater autonomy and accountability;
- A records management system has been designed and implemented;
- Audit charter and audit programme and planning formats have been designed and implemented;
- A debit card system has been designed and implemented;

The number of outstanding imprests has been reduced to a fifth, and imprests are retired in line with the financial regulations.

The efforts to put the plan in motion have lagged behind because the government is involvement in developing some of its components such as the integrated financial management system, which is deemed to be an expensive venture. Further, the response to the auditor general’s queries has not been timely, and monitoring and evaluation of the procurement processes to ensure proper governance has not matched the goals. By involving CSOs in activities, GHIs have influenced their role in advocating for beneficiaries and to some extent in holding the government accountable in certain areas.

In both Tanzania and Zambia most GHIs have promoted civil society and community participation through concrete project funding or involving them in management mechanisms such as country coordinating mechanisms (CCM). The has resulted in improved community awareness on priority health problems, diversification of providers, and expansion and diversification of national actors engaged in promotive, preventive and curative health services. However, these efforts are not matched with an increase in the regulatory and quality control functions of the national authorities.

### Financial management

In Tanzania, the GHIs’ stringent requirement for financial accountability and timely disbursement of funds has forced them to develop and depend on their own systems, which by default has weakened the national financial systems. This is because there is a tendency to use the same human resources that the health sector relies on, but these are already limited and overburdened. The stakeholders felt that the GHIs had not used their financial power to end corruption in Tanzania, citing cases where evidence of embezzlement of GHI funds was available, e.g. in a GFTAM case, but action had not been taken to deal with the culprits.

The GHIs in Zambia have influenced financial management at the national level through participating in the development of the governance and management plan, but these efforts have concentrated on the central level with little attention on the sub-national level. The government is working to introduce new systems, but these are very expensive and have little support from the GHIs.

### Health Information Systems

In Tanzania, GHI support helped make substantial improvement to the health information systems. Furthermore, the mother Health Management Information System, on which all the GHIs depend, has been used to develop other specific GHI health information systems such as the antiretroviral (ART) patient tracking and human resource information systems. However, the longstanding problem of poor data use for planning and decision-making has not seen similar positive changes in Tanzania.

Similarly, interest from the GHIs and donor partners to strengthen the health information systems in Zambia has been high. A partnership between the European Union and GFTAM supported the development of the web-based health information systems in the country.

### Human Resources for Health

Most GHIs in Tanzania have and continue to support pre-service and in-service training of HRH, as well as incentives such as housing and top-up allowances. According to the respondents, however, this support is not comprehensive enough since it does not cover the whole HRH spectrum from their production through their recruitment to their retention.

GHIs were regarded to have contributed to the distortion of the HRH market, which has resulted in internal staff attraction to some of their programmes such as pay top-up, and external migration from the government to nongovernmental organisations, which offer better remuneration. The differential payment arrangements from the GHIs have enhanced the culture of working for what is paid, as one respondent noted,

*Since the advent of GHIs we have seen a lot of health workers leave the government to work for these initiatives … even those who are still in the public sector prefer to work in their projects because there are always additional incentives.* (MoH officer, Tanzania)

The GHIs in Zambia have supported some important initiatives for HRH, given their dire situation, including capacity building, e.g. for clinical specialists, and support of HRH retention schemes and pay for performance initiatives. However, GHI support for HRH has been limited by inadequate staffing, the limited capacity of some of the existing HRH, and government restrictions on salary payments, and other limitations such as task-shifting policies.

### Medicine and technology

The GHIs in Zambia have made a huge contribution to the availability of medicines and other health essentials. This has been possible through facilitation of direct purchases of drugs and vaccines, strengthening of supply chain management systems, support of strategies to reduce stockouts, and availing flexible funds for the purchase of drugs when needed. However, GHI efforts have been constrained by the huge size of the country and the under-functioning logistics system.

### Service delivery

GHIs have been instrumental in the improvement of service delivery for some key diseases like HIV, tuberculosis and malaria. This has been through their support to the various areas of the health system. GHIs have increased access to health services through working with nongovernmental organisations and CSOs, which have access to the beneficiaries such as people living with HIV/AIDS, and also work in remote areas. Further, GHIs have helped to build the capacity of some key staff necessary for the delivery of services such as HIV counselling and testing, as well as to develop or improve the infrastructure required for delivering some of the services, such as medical laboratories. This has made it possible to deliver and integrate other related services such as cervical cancer screening. But these efforts have been restrained by inadequate HRH capacity, inconsistencies across the GHIs, poor support systems and high staff turnover. One of the negative aspects is the fact that these efforts are limited to a few diseases like HIV, malaria, tuberculosis and vaccine-preventable diseases and do not address other equally important issues such as maternal and child health and non-communicable diseases.

According to the respondents and available data, service delivery for specific diseases such as HIV, tuberculosis and malaria had improved tremendously in Tanzania. But the respondents did not regard these services as sustainable since they mostly depended on GHI funding, which was shrinking all the time. There were concerns that the GHI approach of focusing on selected diseases contradicted Tanzania’s health for all policy and exacerbated equity gaps in service delivery. Additionally, GHI interventions do not address the downstream issues that are the root causes of the diseases they focus on, so they were viewed as superficial and non-holistic.

#### SWOT (strength, weakness, opportunity and threats) analysis of GHIs

- Strengths
  - In Tanzania, GHIs were perceived as committed and aligned with the health sector’s strategic plan. Likewise, the main strength of the GHIs in Zambia is that they are aligned with the government’s policy requirements. This has facilitated the attainment of some important goals by Zambia such as the MDGs. The respondents recognised that GHIs had helped raise the profile of some neglected diseases and health areas. An additional advantage associated with the GHIs in Zambia was their ability to mobilise funds for the government.
- Weaknesses
  - The respondents believed that efforts to coordinate and harmonise GHIs in Tanzania had not been successful. Some GHIs such as GFTAM and PEPFAR were regarded as particularly difficult in this regard. Further, these efforts were mainly directed and concentrated at only the planning level. The respondents lamented the duplication of activities at the implementation level for example in the running of workshops, which overwhelmed the districts. Also the GHIs’ failure to integrate the existing SWAp mechanisms was seen as partly responsible for the fragmentation and ineffectiveness of donor coordination in Tanzania, as one respondent noted,
    *One of our biggest challenges is integrating activities at the district level. To date we still operate in silos both from donors and within the Ministry of Health … you can imagine the detrimental effects it has at the district level.* (Development partner representative, Tanzania)
  - One of the main weaknesses in most GHIs is the uncertainty and unpredictability of their funding. The proposal writing processes, despite being intense and time consuming, does not guarantee successful grant funding or funding as per plans. The respondents stated that since most GHIs were not transparent, it was difficult to include them in countries’ plans. The poor harmonisation of GHIs has led to duplication of effort, since local governments exploit this gap to create opportunities for extra personal incomes like per diems from workshops. Duplication of activities has also led to double counting of outcome indicators. The respondents believed that GHIs were limited to an advisory role and rarely influenced legislative processes.
- Opportunities
  - Some good case studies exist that could be used to strengthen GHI functioning. For example, in Tanzania the positioning of a GFTAM liaison officer at the PEPFAR office has resulted in joint planning between these GHIs. The intention and willingness of the GHIs to coordinate activities is an opportunity for the Tanzanian government to initiate similar harmonisation efforts for all GHIs through leadership and guidance. The recent -Big Results Now- renewed effort towards improved performance and accountability is an opportunity to streamline Tanzanian health financing and priorities. Also, Tanzania’s economy is booming, with a rising gross domestic product, providing an opportunity to improve on existing government workforce salaries and incentives for better motivation and performance.
  - In Zambia the separation of roles of the MoH and the Ministry of Community Development, Mother and Child is an opportunity for better planning and implementation of comprehensive interventions that also tackle the social determinants of health. Zambia’s gross domestic product also has improved, moving the country from a low to a middle income nation, which means that GHIs will call for co-financing of programmes by the government for sustainability.
- Threats
  - The biggest threat to GHI functioning and return on investment is the poor status of the workforce through which they implement their interventions. The health workforce in Tanzania’s public sector is generally demotivated owing to poor pay and incentives, and consequently many workers use large portions of their work time on activities to supplement their income. This has led to corruption; poor performance, customer care and service delivery; duplication of activities and nepotism.
  - The existing GHI approaches in Zambia will continue to distort the planning and implementation of activities. At the district level these approaches have had dire effects on adherence to plans owing to the unpredictability of funding.

## Discussion

GHIs have been instrumental in supporting national strategic plans of both Tanzania and Zambia, where they have made a remarkable contribution towards the fight against major diseases such as HIV, tuberculosis and malaria. However, a number of challenges exist related to their governance, coordination and contribution to health systems strengthening.

There is no doubt that in both Tanzania and Zambia financial resources and technical assistance from GHIs have been remarkable [19]. The two two case studies show that several GHIs are supporting the countries’ health programmes. The positive aspects of their contribution relate to their alignment with country priorities, use of national health information systems and improved access of health interventions for the programmes that support.

The negative elements include weaknesses in their coordination as evidenced by their use of their own structures as opposed to SWAp mechanisms. Although GHIs have contributed to the strengthening of health systems, this has not been comprehensive, and in some cases they have caused distortions in the systems. The challenges to the planning of their activities have been related to the lack of comprehensive information and concerns about unpredictability of funding.

GHIs have been the subject of criticism [5, 6, 20, 21]. Literature from their early years cites discrepancies between their objectives and country priorities [6]. In particular, there was concern about the alignment of GHI objectives with those of the strategic plans of various countries across the African Region [6, 22]. From these two case studies, it is unmistakable that GHIs have improved their alignment with national health priorities. Now most of the GHIs in Tanzania and Zambia are guided by either the national development plans or the health strategic plans. The implementation of GHI interventions also uses existing health systems.

This study and other literature show, however, that GHI processes such as selective and restrictive funding for specific areas compromise their alignment with national priorities [4, 20, 23]. Also, as noted in our study, harmonisation between GHIs and other health development partners in both countries is weak. Most of the GHIs do not have a permanent representative or office in the countries to attend coordination meetings on health policy issues or to participate in other processes such as strategic planning and technical discussions. This has led to duplication of efforts and has been the source of an additional burden for the countries [4, 23]. Previous studies also have alluded to the poor harmonisation between GHIs and donors [8, 23]. One such example from the literature was the failure of the World Bank to use the Three Ones’ principles with other partners, which was a prerequisite for the Multi-country HIV/AIDS Program review [24]. Instead of using one strategic framework, one national authority and one monitoring and evaluation framework, the countries are often been burdened with extensive and complex procedural and reporting requirements [8, 20, 23]. This was observed in our study too.

GHIs have a tendency to set up their own coordination mechanisms and a committee that may overrule national level authorities. Our study noted this. Other authors have made similar observations, such as Lancet [20] who claims that GHIs have at times overridden the policy-making authority of UN systems through bypassing them and working directly with countries. Additionally, the involvement of government officials in GHI activities removes them from actively participating in planned government activities and might negatively affect the leadership and stewardship of those activities. Similarly, the high transaction costs for countries with scarce health technical staffing associated with the increased reporting modalities, separate accountability systems and the multitude of meetings hamper implementation of other priority activities [4, 23, 25].

In Tanzania and Zambia several GHIs give priority to health system strengthening explicitly referencing the WHO health systems building blocks. However, there are gaps with that approach [2, 4, 23]. Our study found that none of the GHIs adopted a holistic approach to health systems strengthening, but most of them focused on the main health systems bottlenecks that hampered the attainment of their specific objectives. Some GHIs have addressed the HRH challenges such as those relating to pre- and post-training and staff motivation but little attention has been given to the production side [4, 10, 25–27]. The selective approach to interventions by either area or disease was seen also with health information systems, monitoring and evaluation processes and data quality assurance. Renewed attention and support from GHIs have helped improve procurement and supply management systems, as evidenced in our study and other literature [4]. This support was perceived as creating parallel and unsustainable systems with a tendency to bring in new and foreign staff rather than strengthening the existing country capacity. Further, our study and available evidence indicate that the service delivery activities focused on some specific areas such as HIV/AIDS with interventions such as introduction of standards for treatment and prevention, renovation or building of health infrastructure for specific areas, improvement of laboratory equipment and provision of medicines [4]. This was perceived by respondents in our study as exacerbating inequity in service delivery. Such interventions are not sustainable and could even threaten efforts already made by the countries to improve their health services coverage.

### Study limitations

This study has two country case studies from Africa. Inherent in any case study that is meant to understand the dynamics of an issue is the fact that the extent to which the findings can be generalised is debatable. However, this study has shed light on some of the existing characteristics of GHIs and their operations, providing lessons for countries with similar contexts as Tanzania and Zambia.

## Conclusion

GHIs are important funders for health in low and middle income countries. To optimise the return on investment by GHIs, there is need to improve their governance at the country level. Coordination of GHIs by a single mechanism under the leadership of a national authority could help the countries to tackle existing fragmentation and duplications. Institutional reforms in this regard should be prioritised, building on lessons from the countries using SWAp mechanisms. To reduce transaction costs, there is need for coordination and integration of GHI procedures. A unified procedure under a government authority is recommended. Each country’s health authorities need to be in a position to negotiate on each project aiming at comprehensively strengthening their health systems to achieve universal coverage.

## Abbreviations

CSOs, civil society organisations; GFTAM, Global Fund to Fight AIDS, Tuberculosis and Malaria; GHIs, global health initiatives; HHA, Harmonization for Health in Africa; HRH, human resources for health; HSS, health systems strengthening; MDGs, Millennium Development Goals; MoH, Ministry of Health; PEPFAR, US President’s Emergency Plan for AIDS Relief; PMI, US President’s Malaria Initiative; SWAp, Sector-wide Approach; WHO, World Health Organization

## References

[1] Carlson C (2004). Mapping Global Health Partnerships: What they are, What they do and Where they Operate.

[2] Nervi L (2007). Mapping a sample of global health partnerships: a recount of significant findings. In.

[3] Smith O, Gbangbade S, Hounsa A, Miller-Franco L (2005). Benin: System-Wide Effects of the Global Fund: Interim Findings.

[4] World Health Organization Maximizing Positive Synergies Collaborative Group (2009). An assessment of interactions between global health initiatives and country health systems. Lancet.

[5] Cahill K, Flemming D, Conway M, Gupta S (2003). Global health partnerships: assessing country consequences. High level Forum.

[6] Brugha R, Donoghue M, Starling M, Ndubani P, Ssengooba F, Fernandes B, Walt G. The Global Fund: managing great expectations. Lancet. 2004;364(9428):95–100. 10.1016/S0140-6736(04)16595-1 15234862

[7] Ashley W, Wyss K, Shakarishvili G, Atun R, Don de Savigny D (2013). Global health initiative investments and health systems strengthening: a content analysis of global fund investments. Glob Health.

[8] Biesma RG, Brugha R, Harmer A, Walsh A, Spicer N, Walt G (2009). The effects of global health initiatives on country health systems: a review of the evidence from HIV/AIDS control. Health Policy Plan.

[9] Kapilashrami A, Mcpake B (2013). Transforming governance or reinforcing hierarchies and competition: examining the public and hidden transcripts of the Global Fund and HIV in India. Health Policy Plan.

[10] Warren AE, Wyss K, Shakarishvili G, Atun R, de Savigny D (2013). Global health initiative investments and health systems strengthening: a content analysis of global fund investments. Glob Health.

[11] Grace C (2004). Global Fund country case studies report.

[12] WHO: Everybody business : strengthening health systems to improve health outcomes : WHO’s framework for action. Pg 2. Geneva; World Health Organisation; 2007.

[13] WHO: Everybody business : strengthening health systems to improve health outcomes : WHO’s framework for action. Geneva; World Health Organisation; 2007.

[14] WHO: Everybody business : strengthening health systems to improve health outcomes : WHO’s framework for action. Pg 4. Geneva; World Health Organisation; 2007.

[15] Shiffman J, Smith S (2007). Generation of political priority for global health initiatives: a framework and case study of maternal mortality. Lancet.

[16] World Health Organisation (2015). World Health Statistics 2015.

[17] Global health observatory accessed from http://apps.who.int/gho/data/node.country.country-TZA accessed on the 18th April 2016

[18] Government of Zambia: Ministry of Health Ministry of Community Development Mother and Child Health: Mid-term reveiw of health sector performance. Lusaka-Zambia; Ministry of Health; 2014.

[19] Global health observatory accessed from http://www.who.int/gho/countries/en/accessed on the 18th April 2016

[20] Lancet T (2009). Who runs global health?. Lancet.

[21] Sridhar D, Batniji R (2008). Misfinancing global health: a case for transparency in disbursements and decision making. Lancet.

[22] Stillman K, Bennett S (2005). System-wide effects of the Global Fund: interim findings from three country studies.

[23] Lundstrom T: The impact of Global Health Initiatives and HIV and AIDS programs on the Zambian system - PhD thesis. Sweden: Mid Sweden University; 2012.

[24] Ainsworth M, Vaillancourt D, Gaubatz JH (2005). Committing to results: improving the effectiveness of HIV/AIDS assistance: an OED evaluation of the World Bank's assistance for HIV/AIDS control.

[25] Brugha R, Kadzandira J, Simbaya J, Dicker P, Mwapasa V, Walsh A (2010). Health workforce responses to global health initiatives funding: a comparison of Malawi and Zambia. Hum Resour Health.

[26] Vujicic M, Weber SE, Nikolic IA, Atun R, Kumar R. GAVI: The Global Fund and World Bank Support for Human Resources for Health in Developing Countries; Washington DC: World Bank; 2011. 10.1093/heapol/czs012 22333685

[27] Ssengooba F, Kiwanuka S, Mbona N, Rutebemberwa E, Kirunda B, Buregyeya E (2009). The effects of enhanced availability of funding from Global health intiatives on the distribution, retention and motivation of health workers in Uganda.

